# The Architect Who Lost the Ability to Imagine: The Cerebral Basis of Visual Imagery

**DOI:** 10.3390/brainsci10020059

**Published:** 2020-01-21

**Authors:** Sandra Thorudottir, Heida M. Sigurdardottir, Grace E. Rice, Sheila J. Kerry, Ro J. Robotham, Alex P. Leff, Randi Starrfelt

**Affiliations:** 1 Icelandic Vision Lab, Department of Psychology, University of Iceland, 102 Reykjavik, Iceland; sdt9@hi.is (S.T.); heidasi@hi.is (H.M.S.); 2 Cognition and Brain Sciences Unit, University of Cambridge, Cambridge CB27EF, UK; Grace.Rice@mrc-cbu.cam.ac.uk; 3 Institute of Cognitive Neuroscience, University College London, London WC1N3AZ, UK; sheila.kerry10@googlemail.com (S.J.K.); a.leff@ucl.ac.uk (A.P.L.); 4 Department of Psychology, University of Copenhagen, 1726 Copenhagen, Denmark; jer@psy.ku.dk

**Keywords:** visual imagery, stroke, posterior cerebral artery, aphantasia, prosopagnosia, visual perception

## Abstract

While the loss of mental imagery following brain lesions was first described more than a century ago, the key cerebral areas involved remain elusive. Here we report neuropsychological data from an architect (PL518) who lost his ability for visual imagery following a bilateral posterior cerebral artery (PCA) stroke. We compare his profile to three other patients with bilateral PCA stroke and another architect with a large PCA lesion confined to the right hemisphere. We also compare structural images of their lesions, aiming to delineate cerebral areas selectively lesioned in acquired aphantasia. When comparing the neuropsychological profile and structural magnetic resonance imaging (MRI) for the aphantasic architect PL518 to patients with either a comparable background (an architect) or bilateral PCA lesions, we find: (1) there is a large overlap of cognitive deficits between patients, with the very notable exception of aphantasia which only occurs in PL518, and (2) there is large overlap of the patients’ lesions. The only areas of selective lesion in PL518 is a small patch in the left fusiform gyrus as well as part of the right lingual gyrus. We suggest that these areas, and perhaps in particular the region in the left fusiform gyrus, play an important role in the cerebral network involved in visual imagery.

## 1. Introduction

Thinking of a concept, whether it is a flower or a cat or even a unicorn, can bring up vivid, image-like experiences without external visual input. This is generally referred to as visual imagery or mental imagery, although the latter can extend to other senses (e.g., sound, smell, or touch). The basis of mental imagery has long been debated [1,2,3] and there is still uncertainty about its neural underpinnings.

Zeman and colleagues [4] gave the inability to generate mental imagery a name, aphantasia, and described individuals with congenital aphantasia who never had this ability. The loss of mental imagery following brain injury—acquired aphantasia—in individuals who had normal imagery before their injury is also well documented, dating back at least to Charcot and Bernard [5] (but see [6]). However, as noted by Farah [7], cases of acquired imagery deficits can be associated with a wide range of lesions (occipital, temporal, or parietal) in either hemisphere, and no other functional deficits consistently co-occurred with imagery loss with the exception of loss of (visual) dreaming. One plausible reason for this heterogeneity is that mental imagery is not a single phenomenon but can be divided into relatively distinct components, with different underlying anatomy. Some distinguish between a generation process, long-term visual memory, and an inspection process [7], or subsystems such as appearance-based (e.g., shape/color judgment) vs. spatial (e.g., mental navigation/scanning) imagery [8,9] (see also [10]). Supporting this, a meta-analysis of imaging studies showed that while several regions were coactivated during appearance-based and spatial imagery, the former mapped onto the ventral visual stream while the latter evoked specific activity in the dorsal stream [11].

It has been argued that the primary visual cortex (V1) plays a significant role in visual mental imagery [12,13]. Several studies have shown cortical activation in V1 during imagery tasks (e.g., [14,15,16,17,18]) and rTMS (repetitive transcranial magnetic stimulation) targeting V1 can disrupt visual imagery [15]. In addition, individual differences in mental imagery capability covary with differences in V1 surface area [19], V1 functional connectivity [20], and representational overlap between visual imagery and perception in the retinotopic cortex [21]. However, while patients with intact V1 can have severe impairments in mental imagery [22], seemingly intact imagery without a functioning V1 has also been reported [23,24] (see also [25]).

Thus, damage to V1 appears neither necessary nor sufficient for inducing imagery deficits. A review [26] of case studies suggested that extensive left temporal damage is necessary for a visual imagery deficit for object form or color (see also [11]), and more generally that high-level visual areas in the temporal lobe might be particularly important for visual imagery. The fact that patients have been reported to have both high-level visual deficits and selective imagery loss in the same domain (e.g., severe problems in visual recognition and revisualization of faces, [27]), and that actual viewing and visual imagery for particular objects or object categories can evoke a similar pattern of activity in high-level ventral stream regions [21,28,29], is in alignment with the general idea of shared mechanisms between visual imagery and visual perception (for recent reviews, see [30,31]).

Visual imagery and perception however cannot share all mechanisms as there are patients on record with seemingly preserved mental imagery but impaired visual perception [32,33,34,35,36]. For example, case H.J.A. [32] suffered from visual agnosia, achromatopsia, prosopagnosia, alexia without agraphia and topographical impairments. Despite these deficits, H.J.A.’s mental imagery was relatively—albeit not completely—spared. The opposite pattern, impaired visual mental imagery but relatively normal visual perception, has also been reported [37,38]. An example is a patient who had suffered a left occipital and medial temporal infarct. While his visual recognition abilities were generally good, he showed apparent problems in mental imagery such as describing an elephant as having a “tiny waist” and having trouble with verifying sentences that required visual imagery (e.g., “A grapefruit is larger than an orange”) [37].

Here we present patient PL518, an architect who reported almost complete loss of visual mental imagery following bilateral stroke in the areas supplied by the posterior cerebral artery (PCA). His responses on the Vividness of Visual Imagery Questionnaire (VVIQ, ad modum [39]) as well as a range of visuoperceptual tests are compared to three other patients with bilateral PCA stroke, as well as another architect with a large unilateral PCA stroke in the right hemisphere. We also compare the structural images of their lesions. The aim of the study is to: (a) describe the correspondence between the perceptual and neuropsychological profile of PL518 compared to the other patients, and (b) to delineate cerebral areas that are uniquely affected in the aphantasic patient and could thus play a fundamental role in the generation of visual imagery.

## 2. Materials and Methods

### 2.1. Participants

Patient PL518 and four other patients participated in this specific study. All were recruited as part of a larger study of PCA stroke (the Back of the Brain (BoB) project, described in [40]). 46 controls were included in the BoB project. All participants provided written, informed consent, and the project was approved by the ethical committees of Manchester (North West Research Ethics Committee; MREC 01/8/094) and UCL (London Queen Square Research Ethics Committee, UCL; 16/EM/0348). See Table 1 for demographics and background data for the included patients and controls. Additional background data as well as raw scores on the perceptual and neuropsychological tests can be found in Table S1.

PL518 suffered a bilateral PCA stroke 35 months before the current investigation. At that time, he had corrected to normal visual acuity and a slight visual field defect primarily affecting the parafovea of the upper left quadrant (see Table S1 for acuity and visual field data for all participants). He reported problems with seeing colors following his stroke and scored outside the normal range on a formal test of color perception (Farnsworth D-15 [45]). His intermediate vision (assessed with subtests from the L-POST [46]) was largely uncompromised, except for difficulties with figure-ground segmentation. His basic response time (RT) to visual stimuli (test described in [40]) was unaffected, and his auditory digit span forwards and backwards (WAIS-IV UK [44]) were within the normal range. PL518 reported severe problems in face recognition following his stroke and volunteered that he had problems recognizing his own face in the mirror. He also reported increased problems in finding his way around. Neuropsychological testing showed a clear deficit in face recognition affecting learning of new faces as well as judgment of familiarity and recognition of famous faces. In contrast, he performed within the normal range on several tests of object recognition, including perceptually challenging tests, with the notable exception of a memory test for houses (Cambridge House Memory Test, [47]) designed as an equivalent to the Cambridge Face Memory Test [48]. His word recognition accuracy was well within normal range, while response times in reading out loud and the effect of word length on RT was slightly but significantly elevated compared to controls.

In the context of the BoB project, PL518 spontaneously reported that his visual imagery was “gone” following his stroke, which led us to contact four additional patients either with similar lesions (bilateral PCA stroke) or a similar background (architect) for a follow-up interview about their visual imagery. The lesion location, size, and time since injury for these patients, as well as other background characteristics, are presented in Table 1 along with summary data from the control group.

### 2.2. Visual Imagery

As PL 518 was the focus of the study, a long and in-depth interview was also carried out about his visual imagery before and after his stroke. In order to get more information about his ability to store visual information in his mind, he was asked to carry out the Rey–Osterrieth Complex Figure Test [49] at the end of the interview. The other patients did not complete this test.

All five patients were asked to complete a version of the Vividness of Visual Imagery Questionnaire (VVIQ-modified, [39]), that is a modified version of the VVIQ [50]. VVIQ-modified has 16 items where participants are to imagine various scenarios (e.g., a relative or friend, a rising sun) and rate the vividness of their visual image on a five-point Likert scale where 1 indicates no image at all and 5 indicates that the image is perfectly clear and vivid. Scores on the VVIQ-modified can range from 16 to 80, with 16 representing the lowest possible imagery score and 80 the highest possible imagery score. Various versions of the VVIQ have been validated, and the questionnaire has been shown to be a valid psychometric tool for measuring the vividness of visual imagery with both high construct validity and internal consistency reliability [51,52,53]. PL518 and PM024 performed the questionnaire in the lab while the other three patients were interviewed over the telephone. The four control patients were also asked four general questions about their visual imagery, to compare with the interview of PL518. They were asked to answer the following questions with yes, no, or don’t know: (1) can you imagine things visually in your mind? (if no: do you sometimes experience brief flashes of imagery?); (2) would you say that your memories have a visual aspect to them in your mind?; (3) do you see visual images in dreams?; and (4) has your visual imagery changed following your stroke? The normal controls did not perform the visual imagery questionnaire.

### 2.3. Neuropsychological and Experimental Tests

The BoB project is a comprehensive neuropsychological and imaging project investigating perceptual deficits following posterior brain injury [40]. A main aim of the overall project is to compare patient performance with faces, objects, and words. The main findings of the project are not yet published (paper in preparation, [54]). Here we report data from the five included patients and controls on tests and experiments selected to be comparable across categories for faces, objects, and words, and these are briefly described below. These experiments, as well as all other tests included in the project, are described in full in [40]. The experimental tests were run on laptop computers with screen resolution of 1366 × 768, or on desktop computers with a screen resolution of 1920 × 1080.

#### 2.3.1. Delayed Matching and Surprise Recognition of Words, Objects and Faces—The WOF Test

This novel paradigm is designed to test immediate and delayed memory for words, objects, and faces (WOF). In the first part (delayed matching), participants were asked to decide whether two sequentially presented images varying in size are the same or not. There were 48 trials for each stimulus type and both accuracy and RTs are measured. The second part (surprise recognition) followed after a short break (where participants performed an unrelated task, the Farnsworth D-15). Here, participants were asked to decide whether they saw the presented stimuli in the delayed matching task or not. There were 12 trials, and accuracy and RTs were measured. In total, 12 measures were derived from this task: 2 metrics (accuracy and RT) * 3 stimulus types (words, objects, faces) * 2 paradigms (delayed matching and surprise recognition). See [40] for a more detailed description.

#### 2.3.2. Familiarity Decisions

Familiarity decision tasks were run for faces, objects, and words. For faces, participants were asked to decide whether a presented face was famous or not (80 trials in total). For objects, we used a 72-trial version of a well-studied object decision task [55], presenting line drawings of real objects and chimeric non-objects. Participants were asked to decide if the picture represents a real object or a non-object. For words, we used a lexical decision task with 60 trials, where participants were asked to decide whether the presented letter string represented a real word or a pseudoword. For all three familiarity decision tests, both accuracy and RTs for correctly categorized familiar items (famous faces, real objects, real words) were analyzed.

#### 2.3.3. Naming of Familiar Items

Tests of picture naming (line drawings), face naming (famous faces) and word reading (regular words). For pictures and words, both accuracy and RTs for correctly named items are analyzed (a voice key was used for RT measurement). For famous faces, only accuracy is recorded as measuring RTs in face naming tasks is complicated by participants making other verbal responses than names (e.g., “it’s that guy from the Parliament…”).

### 2.4. Structural MRI: Lesions

Structural brain imaging data were acquired from all subjects. Structural scans were acquired on two 3T Phillips Achieva scanners with 32-channel head-coils and a SENSE factor of 2.5 in London and Manchester. A high-resolution T1 weighted structural scan was acquired for spatial normalization, including 260 slices covering the whole brain with TR = 8.4 ms, TE = 3.9 ms, flip angle = 8 degrees, FOV = 240 × 191 mm^2^, resolution matrix = 256 × 206 and voxels size = 0.9 × 1.7 × 0.9 mm^3^. Automated outlines of the area affected by stroke were generated using Seghier et al.’s modified segmentation–normalization procedure [56]. Segmented images were smoothed with an 8mm full-width half maximum Gaussian kernel and submitted to the automated routines for lesion identification and definition modules using the default parameters. The automated method involves initial segmentation and normalizing into tissue classes of grey matter, white matter, cerebro-spinal fluid (CSF), and an extra tissue class for the presence of a lesion. After smoothing, voxels that emerge as outliers relative to the normal population are identified and the union of these outliers provides the “fuzzy lesion map”, from which the lesion outline is derived. The generated images were used to create the lesion overlap maps.

## 3. Results

### 3.1. Visual Imagery

In the clinical interview, PL518 reported an almost complete absence of visual imagery following his stroke. This was in stark contrast to his (in his own opinion) above average ability for visual imagery before his stroke that he had relied upon in his work as an architect. He said: “Before, my visualization abilities were pretty impressive. At my work, I could visualize and remember things that most people had not thought about. I would be sitting there and I would say, well, you can’t do X, Y and Z, because you’ve got this happening here and there. Now I have to look at the drawing and work my way through it.” During the interview, he also described how it had felt to do a mental rotation task: “I cannot do it as quickly or the same way as I would have done before my stroke. Before, bang, I would just know the answer. Now it is a much more conscious process. It’s almost as though I physically am trying to move things inside my head.” He was then asked whether his difficulty with mental rotation affected his ability to work as an architect, to which he responded: “Well I just do everything on the computer. That is one of the advantages of us using computers for these sorts of thing nowadays. You can see the stuff happen.” He also described how he is just about able to imagine very simple shapes, but this is done using something akin to motor or spatial imagery and he struggles to imagine more than one shape at a time: “If I tried to visualize shapes like a square, pyramid or sphere lined up next to each other, and I try and focus with a kind of spotlight on the corner of one shape, I can mentally trace a line around the shape. But as soon as I focus on one shape, the others disappear.” When asked if he could imagine an elephant, he seemed to mostly think of the abstract concept of an elephant: “I can think of elephants, iconic elephants like Babar or Elmer, but I can only visualize bits of them. It’s almost painful.” When asked to describe the place he stayed during his last holiday and its surroundings he provided few very vague details about a couple of the bars from the street they had lived on, and he apparently did not visually imagine himself there: “I am recalling almost like a list. I do the same when going somewhere. I have to remember a list”.

**Figure 1:**
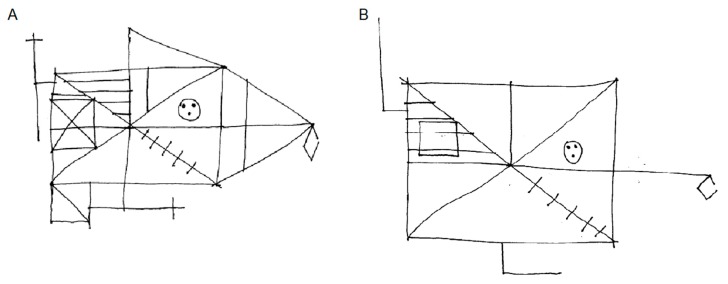
PL518′s performance on the Rey Complex Figure Test. (**A**): Copy. (**B**): three-minute recall.

PL518′s copy and retention of the Rey figure are shown in Figure 1. The drawings were scored for accuracy according to the Taylor’s (1969) method described in Spreen and Straus (1991). 35/36 points were given for the copy and 18/36 for the three-minute recall. While these scores are within the normal range, one could have expected patient PL518, with his background as an architect, to have adopted a more structured approach to drawing the figure in the recall condition. This drawing not only lacks many details but also some of the core elements. Also, some of the included elements are placed incorrectly.

PL518′s complaints regarding his visual imagery were also clearly reflected in his responses on the VVIQ-modified where he scored 18 (i.e., a mean score of 1.13 per item), corresponding to minimal imagery [39]. None of the other patients reported any changes in the nature or vividness of their visual imagery following their strokes–neither in the VVIQ-modified nor the general questions; they all responded yes to the first three general questions about being able to see images in their minds, and no when asked if their visual imagery had changed following their stroke. Their respective scores on the VVIQ-modified were: PL502: 49 (mean: 3.06); PL545: 53 (mean: 3.31); PM006: 72 (mean: 4.5); PM024: 76 (mean: 4.75). See Appendix A Table A1 for the patients’ responses to the individual questions.

### 3.2. Neuropsychological and Experimental Tests

For the accuracy measures, PL518 is clearly impaired with faces, and shows a deficit (performing more than two standard deviations (SDs) from the control mean) on most individual face measures. He performs within the low–normal range on the object tests and is clearly on level with controls in the tests with word stimuli. For the RT measures, PL518 shows a deficit on most face measures (note that his RTs in the surprise recognition test may not be a good indicator of severity, as his accuracy in this test was very low). He responds with latencies within the normal range on the object tests but shows elevated RTs in the lexical decision and word reading tests.

**Figure 2:**
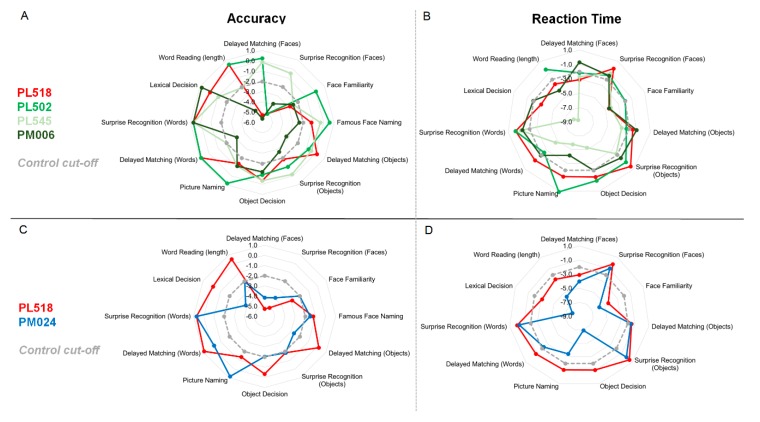
Radar plots showing the results of PL518 (in red) and the other patients on all the included measures of object, word, and face recognition. Numbers denote z-scores based on the control means and SDs for the respective tests. Impaired performance (>−2 SDs from the control mean) is marked by the dotted grey line, and scores closer to the center are more impaired (represents lower accuracy and slower RTs). Left panel (**A**,**C**) shows accuracy, right panel (**B**,**D**) shows RTs. Upper panel (**A**,**B**) shows PL518 vs. PM024 (architect with right hemisphere lesion). Lower panel (**C**,**D**) shows PL518 vs. the other bilateral patients. See individual radar plots comparing PL518 individually to bilateral patients in Figure S1.

Comparing the neuropsychological profile of PL518 to the other included patients, we find that one or more of them show deficits on the same tests/measures and in the cognitive domain(s) as PL518 (see Figure 2 for an illustration of their cognitive profiles on the selected tests, and Table S1 for an overview of test results). A comparison of the neuropsychological profile of the two architects (PL518 and PM024) shows that PM024 (with no aphantasia) shows the same pattern of performance as PL518 on most tests, including measures of face recognition, object recognition and word reading. Indeed, there is no measure on which PL518 shows a clear deficit, where PM024 is clearly within the normal range (see Figure 2). The key difference between the two patients, then, is in the measure of their visual imagery. Comparing PL518 to the three other bilateral patients (Figure 2 and Figure S1), again there is no domain where PL518 is clearly impaired where the other patients are consistently within the normal range. In comparison to the three bilateral patients too, then, the key difference is in visual imagery.

### 3.3. Lesion Localisation

**Figure 3:**
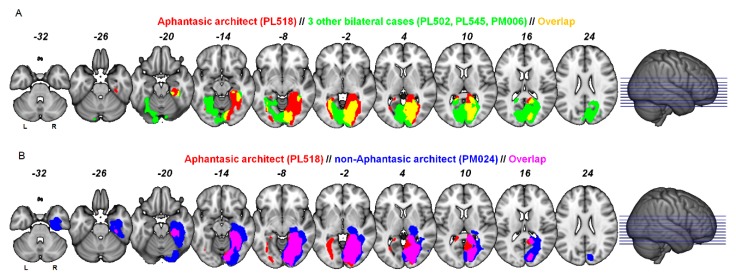
(**A**) shows lesion overlap between PL518 and the other bilateral patients. (**B**) shows lesion overlap between PL518 and PM024. Left hemisphere depicted on the left. The lesions are in MNI space overlaid on the MNI152 template. See Figure S2 for individual comparisons of PL518 and the bilateral patients.

PL518′s lesion is most extensive on the right side, including damage to the occipital pole, the lingual gyrus, the whole fusiform gyrus and extending anteriorly to the parahippocampal region. On the left side, the lesion affects only the medial fusiform gyrus and lingual gyrus, while the left occipital pole, and lateral portions of the fusiform gyrus are spared. See Figure 3 and Table 2 for comparisons of lesion localization for PL518 and the other patients.

First, comparing the lesions of PL518 to the architect without aphantasia (PM024) shows that PL518 has selective left hemisphere posterior medial fusiform damage extending medially and anteriorly along the collateral sulcus, and selective right hemisphere damage to the superior medial lingual gyrus. Second, comparing the lesion of PL518 to the three patients with bilateral strokes but no aphantasia shows that PL518 has selective damage in the right fusiform gyrus and a portion of the right lingual gyrus, and additional smaller areas of selective damage in PL518 are found in the left fusiform gyrus. Combined, these comparisons reveal only small areas of selective damage in PL518 in the right lingual gyrus and left posterior medial fusiform gyrus.

## 4. Discussion

The present study reports case PL518, an architect who lost his ability for visual imagery following a bilateral PCA stroke 35 months prior to this investigation. We compare his performance across a range of perceptual and cognitive tests and a visual imagery questionnaire with four other PCA stroke patients, an architect with a large right hemisphere lesion and three bilateral cases. PL518′s profile on the perceptual and cognitive tests was similar to other cases with the exception that PL518 reported severe visual imagery problems following his stroke. Lesion profiles were also comparable with the exception that PL518 showed selective damage in the right lingual gyrus and left medial posterior fusiform gyrus. It is tempting to suggest that these are both candidate regions for specific involvement in visual imagery.

However, Bogousslavsky and colleagues [60] described a man whose lingual gyrus was destroyed in both hemispheres, while only the middle third of the fusiform gyrus on the left side was affected. His visual imagery was intact for colors, faces (human and animal) and places (streets). The authors concluded that the fusiform gyrus and underlying white matter, rather than the lingual gyrus, was a principal structure for color integration, face recognition, visuo-verbal processing, and corresponding visual imagery. The fact that the current primary case, PL518, had selective damage to the left fusiform gyrus is also more in alignment with other research indicating that left hemisphere regions are more consistently implicated in generating mental imagery than corresponding right hemisphere regions [4,7,22,24,61,62,63,64,65,66,67,68].

A seeming counterexample comes from de Gelder and colleagues [69]. They described patient TN who had bilateral cortical blindness due to lesions in the primary visual cortices in both hemispheres. The lesion also reached some high-level visual ventral areas, including parts of the left posterior fusiform gyrus. Despite this damage, de Gelder and colleagues [69] argued that TN was able to generate visual mental imagery. However, judging from the lesion reconstruction (their Figure 2), the left medial posterior fusiform might have been at least partially spared in this patient. Also, the imagery tasks used involved a significant motor or action component, and correspondingly TN’s functional activation pattern in the imagery conditions was primarily fronto-parietal.

Fitting with a role of the left fusiform gyrus in visual imagery, some developmental prosopagnosics appear to have functional abnormalities in this region [70,71,72] as well as reduced or absent mental imagery, not only for faces but also for objects and scenes [73]. Barton and Cherkasova [74] examined face imagery in prosopagnosics for featural imagery (questions regarding facial features, e.g., “Who has a wider mouth: Sophia Loren or Ingrid Bergman?”) as well as facial configurations (questions on overall face shape or configuration, e.g., “Who has the more angular face: George Washington or Abraham Lincoln?”). In acquired prosopagnosics, they found that right-sided occipito-temporal lesions affected imagery for facial configuration but not for facial features, while bilateral lesions additionally impaired imagery for facial features [74]. This fits well with the left fusiform gyrus responding more to facial features while the right fusiform gyrus is more involved in configural processing [75]. It is possible that the generation of mental imagery heavily relies on the assembly of separately stored visual features or parts, and that this generation of multipart images specifically taxes left hemisphere regions [37,66]. This is consistent with PL518′s description of the fragmented minimal visual imagery that he possibly still has (e.g., visualizing bits of elephants).

Compared to before his stroke, PL518 seems to make greater use of verbal strategies (e.g., recalling a list). If PL518 still has some mental imagery, it nonetheless mostly seems to be based on an altered strategy which could be described as motor, action-based, or spatial, such as mentally tracing a line around a shape or doing mental rotation by physically trying to move things inside the head. This is reminiscent of patient MX [4] who also reported the loss of the experience of visual imagery as well as an unusual or altered strategy when attempting a mental rotation task, where he needed to match individual blocks and angles perceptually when making his decision.

The two architects, PL518 and PM024, had similar functional deficits, including prosopagnosia, but described vastly different visual imagery (minimal vs. very clear and lively). It is tempting to speculate, therefore, that the additional left hemisphere affection in PL518 contributes significantly to his disruption of imagery. In particular, the small patch in the medial left fusiform gyrus where PL518 has unique damage compared to all the four other patients presents as a good candidate for playing a critical part in the generation of visual mental imagery. While our findings indeed suggest that this region is an important node in the cerebral network underlying visual imagery, other areas, including right hemisphere ventral occipito-temporal areas, left hemisphere areas further anterior in the temporal lobe (see e.g., [74]), more posterior areas in the left occipital lobe, and regions outside of the ventral visual stream are also likely to partake in at least some aspects of visual imagery. For example, while mental imagery generation might mainly depend on structures in the posterior left hemisphere, right parietal regions have been found to be important for spatial comparisons of the contents of visual imagery [76], see also [77]. The right hemisphere could also have some ability to generate visual imagery for overall shape [66], and had we included sensitive measures of configural processing deficits in mental imagery in addition to the VVIQ, it is possible that subtle deficits in PM024 could have been discovered. It is also worth noting that the aphantasic architect PL518 had bilateral damage, while mental imagery generation could possibly be taken over by the right hemisphere in cases of unilateral left hemisphere disruption [76].

The most commonly used questionnaire to measure mental imagery is various versions of the VVIQ [39,50]. The VVIQ has good psychometric qualities and vividness correlates with some other behavioral and neural measures of visual imagery [53,78,79]. These questionnaires do have their limitations, though, as they rely on self-reporting and only measure overall vividness of visual mental imagery. Mental imagery is, however, of a multimodal nature [80] and includes for example smell, touch, sound and taste. Also, there are several different aspects of visual imagery, and in order to capture this more completely, a measure would need to include items specifically for spatial imagery, as well as imagery for colors, objects, places, faces, and even subsets of these such as featural vs. configural face imagery. More fine-grained mental imagery questionnaires and additional behavioral measures that likely rely on mental imagery, such as the clock task [81,82,83], the taller/wider task [66,83], or mental letter construction [84], animal tails test [8], drawing objects from memory [85,86] and the binocular–rivalry technique [87,88], would provide further insights into whether mental imagery deficits are due to a loss of all imagery across modalities, specific loss of visual imagery, or specific loss of subcomponents of visual imagery. Such specific aspects of mental imagery were not directly assessed in the present study.

It is still debated whether imagery and perception may be dissociated, or whether they depend on common networks. In one sense, the current results support the former as some patients with heavy damage to ventral stream areas and associated problems with visual cognition nonetheless appear to have intact visual imagery. Our neuropsychological approach suggests that some ventral stream regions might not be necessary for visual imagery despite containing information on imagined objects [21,28,29,89,90]. On the other hand, the areas specifically associated with PL518′s visual imagery loss are better known for their role in visual perception. A key difference between imagery and perception could however lie in their different network dynamics where imagery is dominated by top-down feedback [21,89,90]; this could even map onto different cortical layers within the same region [91,92]. Even if a region serves both perception and imagery, is it still possible that distinct computations and separable subpopulations of neurons are involved.

It should finally be noted that individual differences in premorbid ability for imagery might play a role in the effects of stroke on these abilities. PL518 reported that his abilities for visual mental imagery had been above average before his stroke. These abilities had enabled him to visualize the spatial and visual attributes of buildings and rooms in rich details and contributed greatly to his achievements as an architect. This fits a general pattern noted by Farah [7] where many cases of acquired deficits in visual imagery involved people whose day-to-day activities had likely demanded visualization. As the normal variability in visual imagery from congenital aphantasia to hyperphantasia becomes better understood, this factor may perhaps help explain variability in the effect of brain injury on visual imagery.

## 5. Conclusions

While several brain regions in both hemispheres are involved in different aspects of mental imagery, our results indicate that the right lingual gyrus and especially the left posterior medial fusiform gyrus are candidate regions for specific involvement in *visual* imagery. These regions were only affected in the aphantasic architect PL518 compared to non-aphantasic patients with comparable cognitive and perceptual deficits.

## Figures and Tables

**Table 1:** Demographic and lesion information for the five included patients and controls, and scores on basic tests. Handedness was measured by the short form of the Edinburgh Handedness Inventory (EHI) [41]; depression was measured with the short version of the Geriatric Depression Scale (GDS-15 [42]). General cognition was screened with the Oxford Cognitive Screen (OCS) [43], and the number of impaired subtests are listed. Digit span forward and backward was measured with the WAIS-IV UK [44] and total scores are listed. Basic motor reaction time (RT) was measured by responding to a bar of light presented horizontally on a screen (test described in [40]).

Participant	PL518	PL502	PL545	PM006	PM024	Controls Mean (SD)
Lesion Laterality	Bilat	Bilat	Bilat	Bilat	R	n/a
Age	52	55	62	67	66	62 (15)
Education (years)	18	17	16	12	16	15 (2)
Gender	M	M	M	F	M	24 F
Handedness (EHI)	−50	100	100	100	100	44 Right
Time Since Stroke (months)	35	20	14	36	10	n/a
Lesion Volume (cm^3^)	52	23	57	24	112	n/a
Geriatric Depression Scale (GDS-15)	0	12	1	3	3	n/a
OCS-Impaired Subtests	0	1	6	1	1	n/a
Digit Span Forward (WAIS-IV max = 16)	10	13	15	11	11	11 (2)
Digit Span Backward (WAIS-IV max = 14)	6	6	8	8	6	8 (2)
Basic Motor RT (ms)	370	439	1195	665	686	398 (73)

**Table 2:** Comparison of regions of interest within the occipital and temporal lobes affected in PL518 compared to other patients. The fusiform gyrus (FG) was segmented into four regions (FG1-4: corresponding to posterior medial, posterior lateral, anterior medial and anterior lateral, respectively) according to Lorenz and colleagues [57]. The occipital pole and the lingual gyrus were defined using a conventional atlas [58], and the parahippocampal region was identified using the images from Bouyeure and colleagues [59]. An x indicates that at least 10% of the corresponding region of interest was affected by a patient’s stroke.

	Patient	PL518	PL502	PL545	PM006	PM024
	Laterality	Bilateral	Bilateral	Bilateral	Bilateral	Right
Left hemisphere	Occipital Pole			x	x	
	FG 1	x	x			
	FG 2		x			
	FG 3	x	x			
	FG 4					
	Lingual Gyrus	x	x	x	x	
	Parahipp. Gyrus		x			
Right hemisphere	Occipital Pole	x		x	x	x
	FG 1	x				x
	FG 2	x				x
	FG 3	x	x			x
	FG 4	x	x			x
	Lingual Gyrus	x		x	x	x
	Parahipp. Gyrus	x	x			x

## Supplementary Materials

The following are available online at https://www.mdpi.com/2076-3425/10/2/59/s1, Table S1. Individual neuropsychological patient raw data. Figure S1. Radar plots comparing individual bilateral patients’ neuropsychological profile to PL518. Figure S2. Individual comparison of bilateral patients’ structural MRI with PL518.

## Appendix A

**Table A1:** Individual responses on the Vividness of Imagery Questionnaire (VVIQ)-modified [39]. Total and mean scores in **bold.**

VVIQ Question	PL518	PL502	PL545	PM006	PM024
1	2	4	4	5	5
2	1	3	3	4	5
3	2	1	2	4	4
4	1	1	4	5	5
5	1	4	4	5	5
6	1	3	4	4	5
7	1	3	3	5	5
8	1	5	4	5	5
9	1	3	2	4	4
10	1	1	2	4	5
11	1	3	2	4	4
12	1	5	3	4	5
13	1	4	4	5	5
14	1	3	4	5	4
15	1	2	4	5	5
16	1	4	4	4	5
**Total**	**18**	**49**	**53**	**72**	**76**
**Mean**	**1.13**	**3.06**	**3.31**	**4.50**	**4.75**
