# Supplementary material for: The Architect Who Lost the Ability to Imagine: The Cerebral Basis of Visual Imagery
Source: Brain Sci. 2020 Jan 21;10(2):59. doi: 10.3390/brainsci10020059 (PMC7071355; doi:10.3390/brainsci10020059)
Supplement: Supplementary file 1 [file brainsci-10-00059-s001.zip › brainsci-677495-supplementary/supplementary figures.pdf]

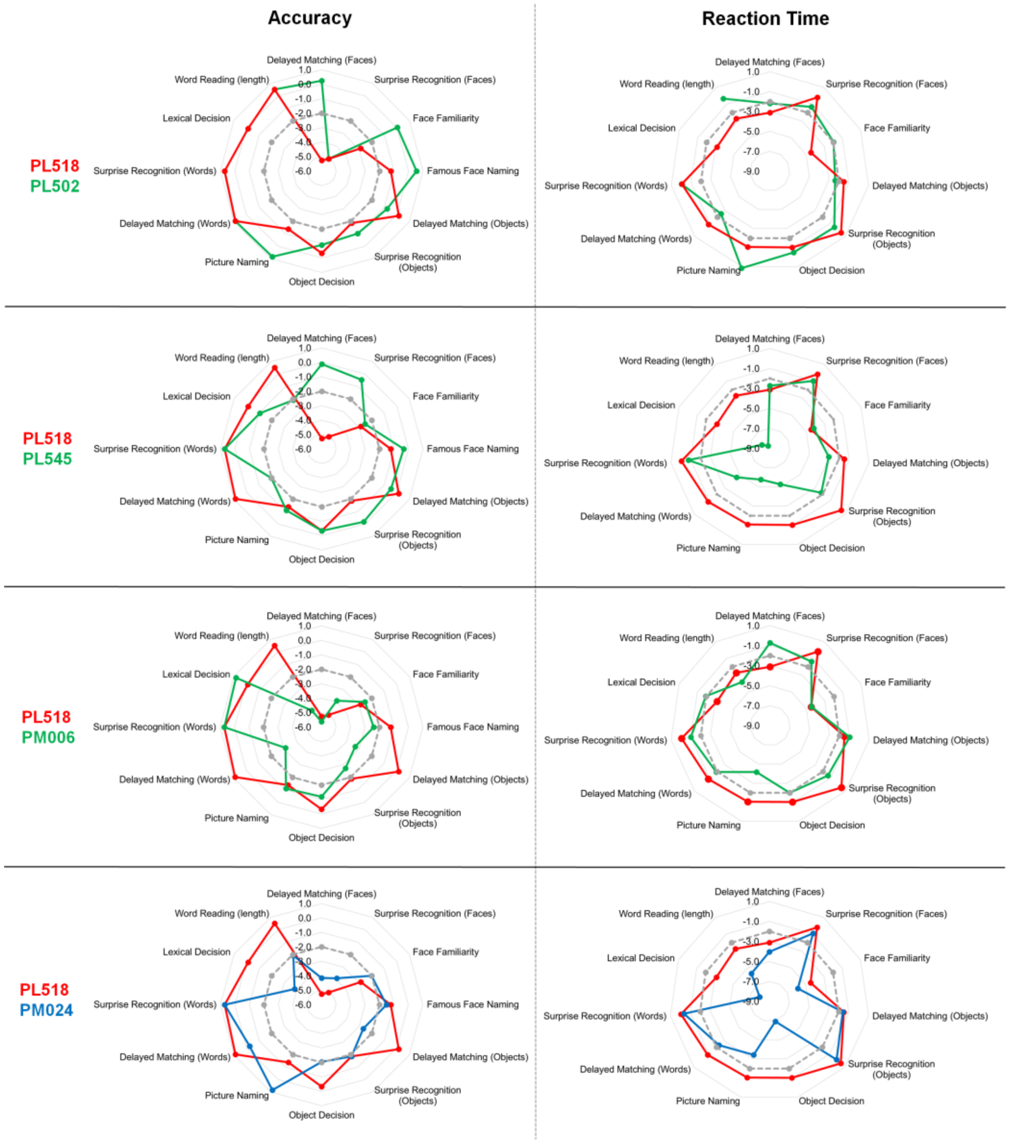

Figure S1. Radar plots comparing individual bilateral patients' neuropsychological profile to PL518.

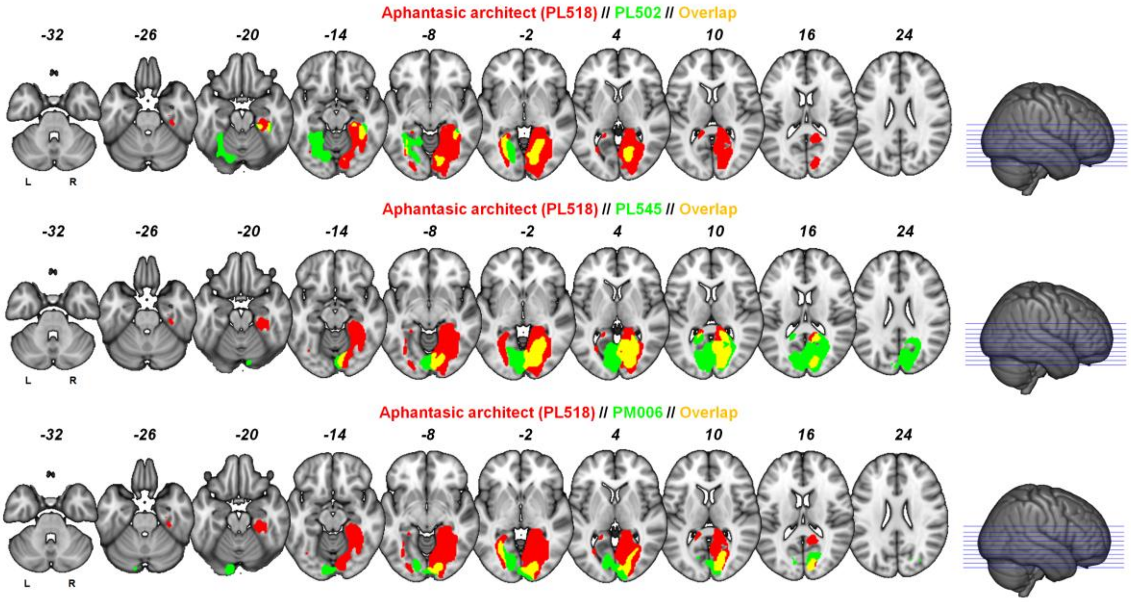

Figure S2. Individual comparison of bilateral patients' structural MRI with PL518.
